# A Closer Look at Potential Underlying Factors Related to Possible Disparity Between Sexes in Delayed Cerebral Ischemia After Aneurysmal Subarachnoid Hemorrhage

**DOI:** 10.3390/jcm14196856

**Published:** 2025-09-27

**Authors:** Michael Veldeman, Tobias Philip Schmidt, Katharina Seyfried, Charlotte Weyland, Karlijn Hakvoort, Tobias Rossmann, Laura Victoria Vossen, Anke Hoellig, Catharina Conzen-Dilger

**Affiliations:** 1 Department of Neurosurgery, RWTH Aachen University Hospital, 52074 Aachen, Germany; 2 Department of Diagnostic and Interventional Neuroradiology, RWTH Aachen University Hospital, 52074 Aachen, Germany; 3 Department of Neurosurgery, Neuromed Campus, Kepler University Hospital, 4020 Linz, Austria

**Keywords:** aneurysmal subarachnoid hemorrhage, delayed cerebral ischemia, sex differences, clinical outcome, infarction volume, treatment responsiveness

## Abstract

**Background**: Aneurysmal subarachnoid hemorrhage (SAH) is over twice as common in females compared to males, who may also experience more severe hemorrhages and worse outcomes. Differences in SAH severity, susceptibility to delayed cerebral ischemia (DCI), and treatment responsiveness may underlie this disparity. This study evaluated sex-based differences in DCI timing, severity, treatment responsiveness, and outcomes after SAH. **Methods**: We analyzed 650 consecutive SAH patients admitted to RWTH Aachen University Hospital (2006–2021). SAH severity was assessed via the (World Federation of Neurological Surgeons) WFNS and modified Fisher scales. DCI-related infarction was defined as new infarcts on CT not present initially or within 48 h post-aneurysm occlusion. Endovascular rescue therapy (ERT) was used for treatment-resistant DCI. Outcomes were assessed at discharge and 12 months using the modified Rankin Scale (mRS). Generalized linear mixed-effects models adjusted for confounders. **Results**: Of 650 patients, 455 (70%) were female. DCI rates did not differ significantly between sexes (41.5% female vs. 36.4% male; *p* = 0.361). DCI-related infarction occurred in 19.4% of patients, with no sex-based differences in infarct volume (median 115 mL; *p* = 0.670) or location. ERT use was similar in females (22.4%) and males (23.9%; *p* = 0.825). Lower age, poor-grade SAH, and higher mFisher scores were associated with DCI and poor outcomes, but sex was not an independent predictor. **Conclusions**: Female sex was not associated with more severe SAH, a higher incidence of DCI, or more severe DCI manifestations. Although small effect sizes may become statistically significant in larger cohorts, our findings indicate that such effects are unlikely to be driven by differences in DCI timing, infarct size, or treatment responsiveness.

## 1. Introduction

Aneurysmal subarachnoid hemorrhage (SAH) is more than twice as prevalent in individuals of the female sex, compared to males across all age groups [1,2,3]. Intracranial aneurysms are more prevalent in women but additionally it might be that aneurysms in women are more prone to rupture compared to men, further contributing to this discrepancy [4,5]. Differences between sexes in SAH severity as well as susceptibility to typical SAH-related complications, such as delayed cerebral ischemia (DCI), have been reported [6]. These disparities usually point to a higher risk of DCI and unfavorable post-SAH clinical outcome, in women compared to men. However, findings from cross-sectional and longitudinal observational studies have been somewhat inconsistent [7]. This may be partially explained by sex-related differences in modifiable risk factors such as smoking, hypertension and alcohol consumption, which vary depending on the population studied.

Since the average age of SAH in women is above the typical age of menopause onset, hormonal changes are often cited as the primary factor contributing to these sex-based discrepancies [8]. However, also, anatomical differences, such as variations in aneurysm location, might contribute to the disparity. Aneurysms in men are more commonly originate from the anterior cerebral artery, whereas in women, they tend to be located more often along the internal carotid artery [9]. This may influence susceptibility to complications like DCI and DCI-related infarction. Moreover, responsiveness to DCI treatment may differ between sexes, affecting outcomes. Even if early outcomes at discharge are similar, differences in rehabilitation potential and long-term recovery may lead to wider gaps over time. These potential mechanisms are not mutually exclusive and could interact in complex ways to influence overall prognosis. Overall, a combination of biological, anatomical, and societal factors might result in differences between sexes in clinical outcome following SAH. Despite sex being an unmodifiable risk factor, understanding sex-specific differences in the risk, timing and intensity of DCI, as well as responsiveness to DCI treatment, could inform more tailored treatment strategies.

Most currently published series fail to assess sex-related differences in rehabilitation potential, as they typically report early outcomes (i.e., discharge or before reaching a plateau in recovery at <12 months) [10]. Furthermore, the effect of the timing of DCI onset, in relation to sex, remains unexplored. The timing of DCI may affect outcome, as early-onset DCI is more resistant to treatment and is associated with worse prognosis [11]. Finally, the degree of responsiveness (or lack thereof) to treatment, indirectly reflected by the need for endovascular rescue treatment (ERT) and the size of DCI-related infarction, has not yet been systematically evaluated.

### Objectives

This study aims to explore whether the timing, intensity, and treatment responsiveness of DCI after SAH differ between men and women, and whether these differences contribute to disparities in clinical outcome.

## 2. Methods

### 2.1. Setting and Participants

All consecutive patients aged 18 years or older who suffered from SAH and presented at RWTH Aachen University Hospital, Aachen, Germany, between January 2006 and December 2021 were considered for inclusion. The causal aneurysm had to be identified using either computed tomography (CT) angiography or conventional digital subtraction angiography.

A prospective SAH registry has been maintained since 2014, with data collection approved by the local ethics committee (EK 22/371 and EK 23/138). Data before 2014 were collected retrospectively from existing electronic medical records. Informed consent was waived by the ethics committee for retrospectively included patients, while consent was obtained from all prospectively included patients. Prospective data collection was registered in the German Clinical Trial Register (DRKS00030505).

Delay in presentation was noted as the number of days between reconstructed ictus (onset of symptoms) and the day of presentation/hospitalization, as delayed presentation can be a driver of worse outcome [12]. Clinical hemorrhage severity was assessed based on the best Glasgow Coma Scale score within 24 h after ictus and stratified according to the World Federation of Neurological Surgeons (WFNS) grading scale [13]. Severity was categorized into good-grade (WFNS 1–3) and poor-grade (WFNS 4–5) SAH. Radiological severity of SAH was graded using the modified Fisher scale (mFisher) and dichotomized into mFisher 1–2 vs. mFisher 3–4 [14].

### 2.2. Study Design and Outcome Variables

This study was designed as a two-group cohort analysis, comparing male and female patients. The aim of the study was to assess differences in clinical outcomes between sexes and to explore potential drivers of disparities in the occurrence and timing of DCI, the responsiveness to DCI treatment as reflected by the need for ERT along the occurrence and size of DCI-related infarction. For this purpose, DCI-related infarction was defined as cerebral infarction on CT scan, not present on initial imaging or imaging performed within 48 h after aneurysm occlusion and not attributable to other causes (e.g., thromboembolism, retractor injury, or edema surrounding intracerebral hemorrhage) [15]. Infarct volume (mL) was calculated using software (Brainlab, Munich, Germany) by two independent assessors. For patient with DCI-related infarction, the involvement of speech areas or the primary motor cortex, was noted based on three-plane CT reconstruction. Because handedness was not routinely documented, speech areas were defined as the posterior section of the left superior temporal gyrus, the left supramarginal gyrus and the triangular and opercular part of the left inferior frontal gyrus. The time point of infarction was estimated by the day after ictus with an available CT scan demonstrating DCI-related infarction.

Neurological outcome was assessed at discharge, and after 12 months. Outcome was classified using the modified Rankin scale (mRS) and dichotomized into favorable (mRS 0–3) or unfavorable (mRS 4–6) outcomes [16]. Outcome assessment before 2014 was performed retrospectively based on existing medical records. As such, blinding of assessors was not feasible, since clinical outcome evaluation required interpretation of available documentation. Since 2014, outcome was assessed by contacting the patient, their next of kin, or caregiver in a structured telephone interview conducted by a blinded assessor.

### 2.3. Treatment Algorithm

Aneurysm occlusion was aimed for early (within 24 h) either via clip ligation or endovascular embolization, after multidisciplinary consensus. Thereafter, patients were surveilled in a dedicated neurointensive care unit. Before 2014, DCI was diagnosed whenever possible based on clinical assessment, ref. [15] or transcranial Doppler (TCD), where vasospasm (mean flow velocity > 120 cm/s) could trigger treatment. Since 2014, patients with an unreliable neurological examination received multimodal monitoring consisting of brain tissue oxygenation (cut-off: <20 mmHg) and/or cerebral microdialysis (cut-off: lactate/pyruvate ≥ 40). Aberrant monitoring measurements could trigger radiological confirmation via perfusion CT scanning (CTP). Radiologically confirmed DCI was defined as territorial or watershed perfusion deficits on CTP with a time to drain > 10 s and mean transit time > 6.7 s. Following clinical, TCD, or radiological confirmation, treatment was initiated.

First-tier treatment consisted of inducing euvolemic hypertension through norepinephrine infusion. Treatment was reevaluated clinically, via CT, or with CTP 6 to 12 h after starting treatment. From 2011 onwards, second-tier ERT was available. The indication for ERT was discussed on an individual basis and implemented following interdisciplinary consensus. Angioplasty was used for proximal vasospasm, while intra-arterial spasmolysis was applied in cases of diffuse vasospasm.

### 2.4. Statistics

Numeric variables were assessed for normality using histograms and quantile-quantile (Q-Q) plots and, where necessary, the Shapiro–Wilk test. Normally distributed numeric variables are reported as mean ± standard deviation and were compared between groups using unpaired *t*-tests. Non-normally distributed data are reported as medians with interquartile ranges (Q1 to Q3) and analyzed using Mann–Whitney U tests. Categorical variables are expressed as proportions (%) and tested using χ^2^ tests.

A difference in cohort sample size between sexes was anticipated. The possibility of propensity score matching (PSM) or inverse probability weighting was considered, depending on the presence and severity of confounding.

Conversely, in order to compare functional outcomes between men and post-menopausal women after aneurysmal SAH, a matching procedure was performed. Post-menopausal women, defined as female patients aged ≥ 50 years based on data for Germany [17], were selected from the study cohort. Eligible male patients aged ≥ 50 years were identified for matching. Matching was conducted using nearest-neighbor matching without replacement based on age, WFNS grade, modified Fisher scale, and aneurysm location (anterior vs. posterior circulation). The matching algorithm was implemented using the Mahalanobis distance for covariate balance optimization.

Over the 16-year inclusion period, two known factors relevant to the prespecified outcome variables changed within the cohort. In 2010, ERT became available for the treatment of DCI. From 2014 onwards, DCI diagnostics were supported by invasive multimodal monitoring (INM). In before vs. after analyses, we previously identified both factors as positive contributors to favorable outcome [18,19]. Therefore, despite having only two levels (before and after), these factors though not of direct interest, represent clustering within the data. A generalized linear mixed-effects model (GLMM) was constructed by introducing predictors based on univariate test results and clinical relevance. The availability of ERT and INM were planned to be treated as both a fixed and random effects when modeling outcomes.

A post hoc power analysis was conducted to evaluate the ability of the study to detect observed differences in the incidence of DCI and DCI-related infarction between sexes. Effect sizes were calculated using Cohen’s *h*.

Statistical significance was defined as a two-sided *p*-value < 0.05. The alpha-level was adjusted according to Bonferroni correction in case of significant results after multiple testing. The study adhered to the Strengthening the Reporting of Observational Studies in Epidemiology (STROBE) guidelines [20].

Statistical analyses were performed, and graphics were created using R (v. 4.4.0; www.r-project.org) in RStudio (v. 2024.12.0+467).

## 3. Results

### 3.1. Demographics

**Figure 1:**
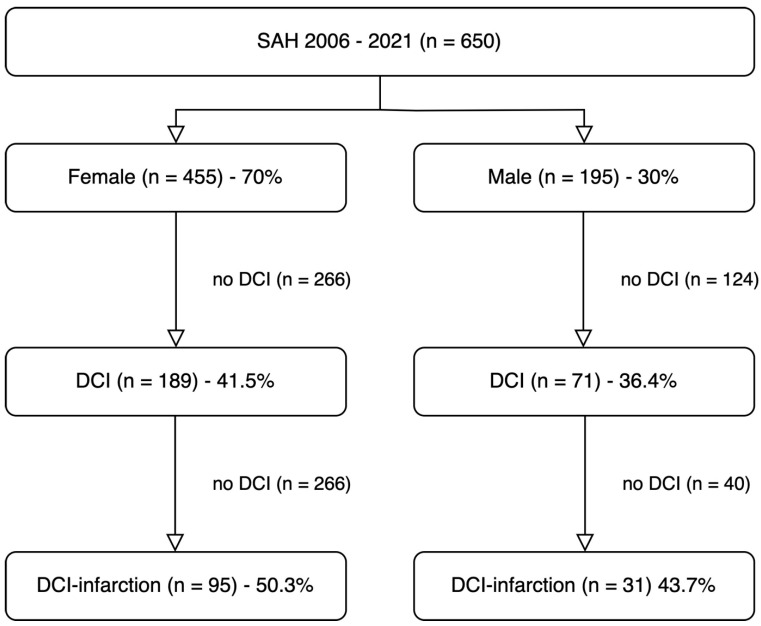
**Flow chart of patient inclusion.** DCI, delayed cerebral ischemia; DCI-infarction, cerebral infarction related delayed cerebral ischemia; SAH, subarachnoid hemorrhage.

Of the 650 included SAH patients, 455 (70%) were female (see Figure 1). Data from 322 (49.5%) patients were collected retrospectively, while data from 328 (50.5%) patients were collected prospectively. The age of women and men was comparable (54.7 ± 13.1 vs. 56.0 ± 13.4 years; *p* = 0.234). Apart from the prevalence of coronary heart disease, comorbidities were distributed equally between sexes. Therefore, propensity score matching or weighting was not performed. In 91 (14%) patients, the initial symptoms did not prompt to seek medical care the same day leading to delayed presentation. Theroff, 62 (9.5%) were female and 29 (4.5%) were male (*p* = 0.767). Females presented with a median delay of 1.5 (1 to 4) days and males with a median delay of 1 (1 to 5) day (*p* = 0.967).

Aneurysm locations differed between female and male patients, driven by a higher number of middle cerebral artery (MCA) aneurysms in females and a higher prevalence of anterior communicating artery (Acomm) aneurysms in males. This difference was corrected for in the multivariate analysis, using the Acomm location (the most prevalent) as the reference category. Aneurysms in female patients were slightly smaller (median maximal diameter of 6 mm vs. 7 mm; *p* = 0.038); however, females were more often carriers of multiple aneurysms. Two male patients in this cohort presented with giant aneurysms (>25 mm).

Relevant shifts in clinical severity (WFNS) and radiological severity (mFisher) were identified when all ordinal levels were considered. However, this did not translate into a significantly higher rate of poor-grade SAH in either sex. Baseline characteristics and hemorrhage severity scores are presented in Table 1.

### 3.2. Occurrence and Timing of DCI

A total of 260 patients (40%) were diagnosed with delayed cerebral ischemia (DCI), typically occurring at a median of six days after aneurysm rupture. The incidence of DCI was similar between females and males, 189 of 455 (41.5%) vs. 71 of 195 (36.4%), respectively (*p* = 0.361). This corresponds to an absolute risk difference of 5.1 percentage points and a not statistically significant relative risk of 1.14 (95% CI 0.92 to 1.41).

**Figure 2:**
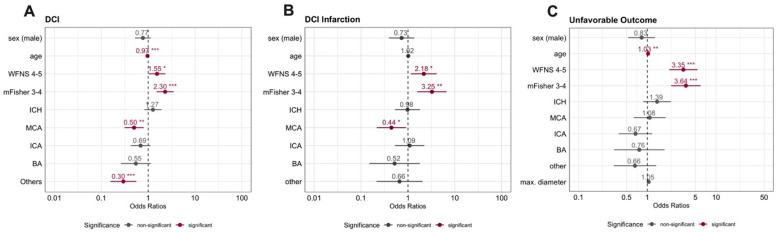
Results of Generalized Linear Mixed-Effects modeling of (**A**) delayed cerebral ischemia (DCI), (**B**) DCI-related infarction, and (**C**) dichotomized clinical outcome after one year, as measured by the modified Rankin scale. Results are in relation to unfavorable outcome (mRS 4–6). Results demonstrate Odds Ratios with respective 95% confidence intervals. Asterisks (*) are used to indicate significance levels as follows: Significance Levels: *** *p* < 0.001, ** *p* < 0.01, * *p* < 0.05, no asterisk: not significant (*p* ≥ 0.05). BA, aneurysm of the basilar artery; DCI, delayed cerebral ischemia; DCI-infarction, cerebral infarction related to unsuccessful treatment of delayed cerebral ischemia; ICA, aneurysm of the internal carotid artery (including the posterior communication artery and anterior choroidal artery); ICH, intracerebral hemorrhage; MCA, middle cerebral artery; mFisher, modified Fisher score for radiological severity in aneurysmal subarachnoid hemorrhage; mL, milliliter; Others, aneurysms located at other locations than those listed; mRS, modified Rankin scale; WFNS, World Federation of Neurosurgical Societies aneurysmal subarachnoid hemorrhage grading scale.

The probability of DCI development over time after aneurysm rupture is plotted in Supplementary Figure S1A and did not demonstrate a different time trend between sexes. The age distribution of female and male DCI cases largely overlapped (see Supplementary Figure S1C). Of the 429 patients for whom endovascular rescue treatment (ERT) was available, 198 (46.2%) were diagnosed with DCI. Induced hypertension failed in 98 (22.8%) patients, requiring subsequent ERT. The need for rescue treatment was comparable between female (22.4%) and male patients (23.9%; *p* = 0.825). In a GLMM, lower age, poor-grade SAH, and a higher mFisher score were associated with the occurrence of DCI. After adjusting for these covariates, as well as differences in aneurysm location between sexes, sex was not significantly associated with DCI. Results are illustrated in Figure 2 and provided in Supplementary Table S2.

### 3.3. Occurrence and Timing of DCI-Related Infarction

A total of 126 patients (19.4%) experienced DCI-related infarction, which occurred at a median of 8 days after aneurysm rupture. Among patients with DCI, infarction occurred in 50.3% of females (95 of 189) and 43.7% of males (31 of 71). This corresponds to an absolute risk difference of 6.6 percentage points and a relative risk of 1.15 (95% CI 0.85 to 1.55), indicating a non-significant increase in the risk of infarction among female patients. The timing of infarction onset after ictus and the age distribution of affected patients were comparable between sexes (see Supplementary Figure S1B,D).

**Figure 3:**
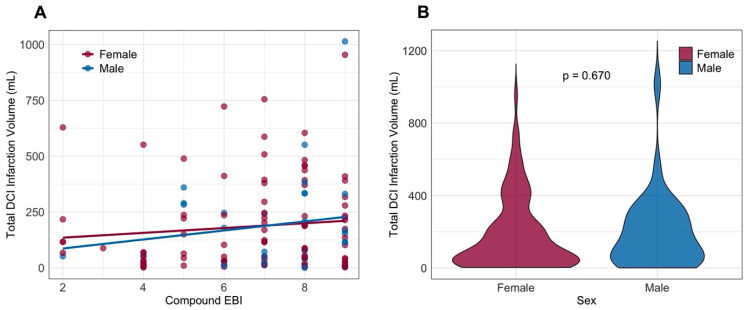
**Volume of DCI-related infarction compared between the female and male sex.** (**A**) Scatterplot of compound early brain injury scoring (WFNS + mFisher) with linear regression trend lines, plotted against infarct Volume (mL) for females (burgundy red) and males (blue). (**B**) Violin plot of infarct volume distribution for females (burgundy red) and males (blue). EBI, early brain injury; DCI, delayed cerebral ischemia; mFisher, modified Fisher score for radiological severity in aneurysmal subarachnoid hemorrhage; mL, milliliter; WFNS, World Federation of Neurosurgical Societies aneurysmal subarachnoid hemorrhage grading scale.

To assess whether a sex-based difference exists in how the severity of early brain injury (EBI) influences responsiveness to treatment (or lack thereof), WFNS and mFisher scores were combined into a composite EBI level. Measured infarction volumes for both sexes were plotted against this composite EBI score in Figure 3A. The plot demonstrates that more severe EBI contributes to larger infarction volumes, but this effect does not appear to be more pronounced in either sex. After fitting linear regression lines, slopes do not differ significantly between sexes (*p* = 0.698). The median infarction volume was 115 mL, and volumes were comparable between female and male patients (*p* = 0.670) (see Figure 3B). Neither the rate, timing, nor size of DCI-related infarction differed between sexes (see Supplementary Table S1). Similar proportions of female and male patients demonstrated infarctions affecting the primary motor cortex or speech areas. In the second GLMM, poor-grade SAH and a higher mFisher score were independently associated with DCI-related infarction. After adjusting for clinical and radiological hemorrhage severity, as well as differences in aneurysm location, sex was not associated with DCI-related infarction. Results of the multivariate modeling are graphically depicted in Figure 2B and presented in Supplementary Table S2.

### 3.4. Recovery After SAH

**Figure 4:**
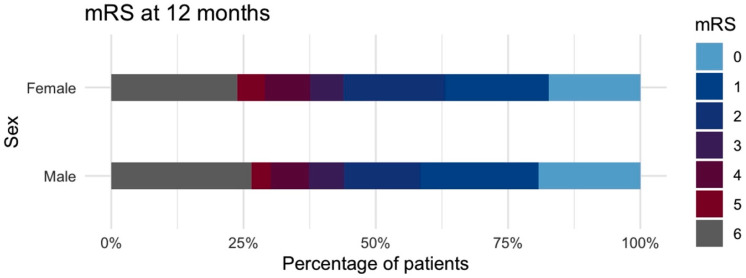
Stacked bar chart of clinical outcomes 12 months after aneurysmal subarachnoid hemorrhage, graded by the modified Rankin scale and split between female and male sex. mRS, modified Rankin scale.

When looking at individual outcome categories at discharge, there are differences in the distribution between female and male patients. This, however, did not translate into a difference in the rate of favorable outcome (females: 40% vs. males: 35.4%; *p* = 0.333). Clinical outcome data after one year was missing for 74 (11.3%) patients. Equally distributed outcome categories were observed after one year. At this time point, 256 (56.3%) female and 104 (53.3%) of male patients, were rated as being in a state of independence (favorable outcome). Clinical outcome at discharge and after one year is presented in Supplementary Table S1 and the latter is graphically depicted in Figure 4. In a third GLMM, higher age, poor-grade SAH, and a higher mFisher score were associated with unfavorable outcome. Results are presented in Supplementary Table S2 and plotted in Figure 2C. After adjustment for these parameters, along differences in aneurysm location, sex did not contribute to one-year clinical outcome.

In a third GLMM, higher age, poor-grade SAH, and a higher mFisher score were associated with unfavorable outcome. Results are presented in Supplementary Table S2 and plotted in Figure 2C. After adjustment for these parameters, along differences in aneurysm location, sex did not contribute to one-year clinical outcome.

### 3.5. Comparison of Post-Menopausal Outcomes

Following matching, the distribution of age, WFNS grade, mFisher grade, and aneurysm location was well-balanced between post-menopausal women and men. A total of 169 matched pairs were included in the final analysis. The proportion of favorable one-year outcomes (mRS 0–2) did not differ significantly between women and men (*p* = 1.000).

### 3.6. Post Hoc Power Analysis

For DCI, the observed effect size was small (Cohen’s *h* ≈ 0.105), corresponding to a statistical power of only 23.3%. To achieve 80% power for detecting such a small effect, a total sample size of approximately 3377 patients (2364 females and 1013 males) would have been required. Similarly, for DCI-related infarction, although the effect size was slightly larger (Cohen’s *h* ≈ 0.13), yet the study’s power remained limited at 15.8%. To identify this difference as significant, with 80% power, an estimated 2256 DCI patients would be needed (1640 females and 616 males). Given the 40% incidence of DCI in our cohort, this would translate to a total of approximately 5640 SAH patients to adequately power such an analysis.

## 4. Discussion

In this observational cohort analysis, more than two thirds of consecutive SAH patients were female. While our findings suggest a higher incidence of DCI and DCI-related infarction in female patients, the effect sizes were modest and did not reach statistical significance. Similarly to previous reports, a higher rate of aneurysms at the Acomm location was noted in men [21]. Additionally, the time point when DCI or DCI-related infarction was diagnosed did not differ between sexes. Responsiveness to DCI treatment was indirectly assessed by looking at the rated of rescue treatment where no differences were observed between female and male patients. Also, the size of DCI-related infarctions proved comparable between sexes.

Reports on sex-related differences in outcome after SAH have shown inconsistent results. An analysis of 161,086 patients with SAH from the US Nationwide Inpatient Sample (2000–2019) found no significant differences in SAH severity scales between sexes [22]. However, female patients were less likely to be discharged to home or a short-term care facility. This result might be driven by the large sample size as actual effect sizes remained small. Cia et al. conducted a retrospective analysis of 283 consecutive SAH patients, of whom 184 (65%) were female [23]. They observed a higher proportion of anterior cerebral artery aneurysms (including Acomm) among males. After matching for age and occlusion modality using PSM, the combined prevalence of DCI and DCI-related infarction was significantly higher in females (27.2% vs. 15.2%). Functional outcome at discharge, showed a higher proportion of poor outcomes (mRS ≥ 2) in females compared to males. Similarly, Han et al. conducted a retrospective cohort analysis of 665 SAH patients, including 424 females [24]. Smoking prevalence was substantially higher among men (65.3% vs. 16%), but clinical and radiological hemorrhage severity was comparable between sexes. After PSM, female sex remained associated with poor 3-month functional outcomes (mRS ≥ 2). Interestingly, even after matching, WFNS and mFisher grading remained significantly different between sexes despite being included as balancing variables.

In another retrospective analysis of 472 SAH patients, the time-based probability of DCI occurrence was assessed, but differences between sexes were not directly compared [25]. A higher rate of clinical DCI was reported in female patients compared to males, though the study did not clarify how DCI was diagnosed in patients for whom neurological examination was not feasible. Despite the difference in DCI rates, functional outcomes at discharge were comparable between sexes.

Finally, in a recent 2024 meta-analysis results from 74 studies examining the effect of sex on DCI, mortality and functional outcomes were generally comparable between sexes, but female patients showed higher odds of developing DCI [26]. Notably, the meta-analysis required studies to define DCI using an accepted clinical definition but did not account for how DCI was diagnosed in unconscious patients.

### Limitations

This data was collected over a large time period, which may have introduced identifiable and subtle changes in treatment strategies that could have affected the assessed outcomes. Two key changes over time were identified a priori: the introduction of endovascular rescue treatment and invasive neuromonitoring. To account for these temporal variations, multivariate analysis was adjusted for these patient clusters by including this factor as both a fixed and random effect.

Approximately half of the data underlying this study was collected retrospectively. While clinical outcome and DCI-related infarction data are likely to be robust even when collected retrospectively, reconstructing the precise timing and occurrence of DCI may be more prone to bias. The heterogeneity in DCI definitions over time (before vs. after 2014) might have introduced misclassification of DCI, which could not be corrected for.

Although propensity score matching (PSM) is often applied in observational studies to reduce confounding, in our cohort baseline characteristics such as age, comorbidities, and hemorrhage severity were already well balanced between sexes. Performing PSM would therefore not have improved comparability but would have substantially reduced the sample size and statistical power. We acknowledge, however, that the female-to-male ratio remained unequal. This reflects the true epidemiology of SAH and was therefore preserved in our analysis. Importantly, the observed absolute risk difference of ~5% was not statistically significant in our sample. However, this difference may warrant further investigation in larger cohorts, as modest effects may become significant with increased statistical power as demonstrated in the post hoc power analysis.

## 5. Conclusions

In our cohort, female sex was not associated with more severe SAH, a higher incidence of DCI, or more severe DCI manifestations. Although small effect sizes may become statistically significant in larger cohorts, our findings indicate that such effects are unlikely to be driven by differences in DCI timing, infarct size, or treatment responsiveness. Post hoc power analyses indicate that sample sizes exceeding 3000 to 5000 patients would be required to detect such differences with sufficient power. This might put into question the clinical significance of these differences.

## Figures and Tables

**Table 1:** Patient-, aneurysm- and hemorrhage-specific data of 650 consecutive patients suffering from aneurysmal subarachnoid hemorrhage, split by female and male sex.

	All	Female (%)	Male (%)	Univariate *p*-Value
	n = 650	n = 455 (70)	n = 195 (30)	
**Demographics and comorbidity**				
age—yrs.—mean ± SD (range)	55.1 ± 13.2 (19–90)	54.7 ± 13.1 (19–87)	56.0 ± 13.4 (19–90)	0.234
BMI—median (Q1 to Q3)	24.9 (22.5 to 27.7)	24.8 (22.3 to 27.7)	25.5 (23.2 to 27.7)	0.353
hypertension—no. (%)	280 (43.1)	199 (43.7)	81 (41.5)	0.708
smoking—no. (%)	195 (30.0)	133 (29.2)	62 (31.8)	0.531
type 2 diabetes—no. (%)	22 (3.4)	16 (3.5)	6 (3.1)	0.969
coronary artery disease—no. (%)	49 (7.5)	27 (5.9)	22 (11.3)	**0.026**
**Aneurysm location—no. (%)**				
Acomm	204 (31.4)	127 (27.9)	77 (39.5)	**0.005**
ICA	137 (21.1)	104 (22.9)	33 (16.9)	0.111
MCA	170 (26.2)	130 (28.6)	40 (20.5)	**0.041**
BA	49 (7.5)	33 (7.3)	16 (8.2)	0.795
others	90 (13.8)	61 (13.4)	29 (14.9)	0.710
posterior circulation	140 (21.5)	99 (21.8)	41 (21.0)	0.994
max. diameter (mm)—median (Q1 to Q3)	6.0 (4.0 to 8.2)	6.0 (4.0 to 8.0)	7.0 (5.0 to 9.0)	**0.038**
multiplicity	163 (25.1)	125 (27.5)	35 (17.9)	**0.048**
giant aneurysms—no. (%)	2 (0.3)	0	2 (1.0)	0.086
**Occlusion modality—no. (%)**				
clipping/endovascular	298 (45.8)/352 (54.2)	212 (46.6)/26 (53.4)	86 (44.1)/109 (55.9)	0.720
**Hemorrhage severity**				
**WFNS grade—no. (%)**				0.055
grade 1	148 (22.8)	93 (20.4)	55 (28.2)	
grade 2	118 (18.2)	91 (20.0)	27 (13.8)	
grade 3	115 (17.7)	78 (17.1)	37 (19.0)	
grade 4	122(18.8)	93 (20.4)	29 (14.9)	
grade 5	147 (22.6)	100 (22.0)	47 (24.1)	
poor-grade SAH (WFNS 3–5)	269 (41.4)	193 (42.4)	76 (39.0)	0.466
**modified Fisher scale—no. (%)**				**0.009**
grade 1	153 (23.5)	101 (22.2)	52 (26.7)	
grade 2	93 (14.3)	68 (14.9)	25 (12.8)	
grade 3	193 (29.7)	151 (33.2)	42 (21.5)	
grade 4	211 (32.5)	135 (29.7)	76 (39.0)	
intracerebral hemorrhage	217 (33.4)	152 (33.4)	65 (33.3)	0.989
acute hydrocephalus	467 (71.8)	333(73.2)	134 (68.7)	0.322
**DCI diagnostics—no. (%)**				
INM available	328 (50.5)	217 (47.7)	111 (56.9)	0.656
PtiO_2_	136 (20.9)	93 (20.4)	43 (22.1)	0.548
CMD	105 (16.2)	69 (15.2)	36 (18.5)	0.988
Dual monitoring	67 (10.3)	67 (14.7)	35 (17.9)	0.376

Acomm, anterior communicating artery; BA, basilar artery; BMI, body mass index; CMD, cerebral microdialysis; ICA, internal carotid artery; INM, invasive neuromonitoring; mm, millimeter; PtiO_2_, brain tissue partial oxygen pressure; Q1, first quartile; Q3, third quartile; SD, standard deviation; yrs., years; WFNS, World Federation of Neurosurgical Societies aneurysmal subarachnoid hemorrhage grading scale. Giant aneurysms refer to aneurysms with a maximum diameter exceeding 25 mm. Significant results are written in bold.

## Data Availability

The raw data of this analysis can be made available by the authors to any qualified researcher upon reasonable request.

## Supplementary Materials

The following supporting information can be downloaded at: https://www.mdpi.com/article/10.3390/jcm14196856/s1, Figure S1: Probability density plot of (A) DCI and (B) DCI-related infarction occurring over time (days) after ictus onset (A & B) or in relation to patient age (C & D). DCI, delayed cerebral ischemia; DCI-infarction, cerebral infarction related to unsuccessful treatment of delayed cerebral ischemia; Table S1: Specific and non-specific intensive care complications along clinical outcome after 6 and 12 months of follow-up; Table S2: Uni- and multivariate analysis of factors associated with delayed cerebral ischemia (DCI), DCI-related infarction and dichotomized clinical outcome after 12 months. Favorable outcome refers to modified Rankin scale 0–3 and unfavorable outcome to modified Rankin scale 4–6. Multivariate analysis is the result of Generalized Linear Mixed-Effect Modelling applying the availability of INM and ERT as random effects.

## Abbreviations

Acomm	Anterior communication artery
CT	Computed tomography
CTP	Computed tomography perfusion scanning
DCI	Delayed cerebral ischemia
EBI	Early brain injury
EVD	External ventricular drain
ERT	Endovascular rescue treatment
GLMM	Generalized linear mixed-effects model
mFisher	Modified Fisher scale
mL	Milliliter
mRS	Modified Rankin scale
PSM	Propensity score matching
Q1	First quartile
Q3	Third quartile
SAH	Aneurysmal subarachnoid hemorrhage
TCD	Transcranial Doppler
WFNS	World Federation of Neurosurgical Societies
