# Supplementary material for: A Closer Look at Potential Underlying Factors Related to Possible Disparity Between Sexes in Delayed Cerebral Ischemia After Aneurysmal Subarachnoid Hemorrhage
Source: J Clin Med. 2025 Sep 27;14(19):6856. doi: 10.3390/jcm14196856 (PMC12525044; doi:10.3390/jcm14196856)
Supplement: Supplementary file 1 [file jcm-14-06856-s001.zip › jcm-3826757-supplementary.pdf]

Supplemental Figures

Supplemental Figure S1

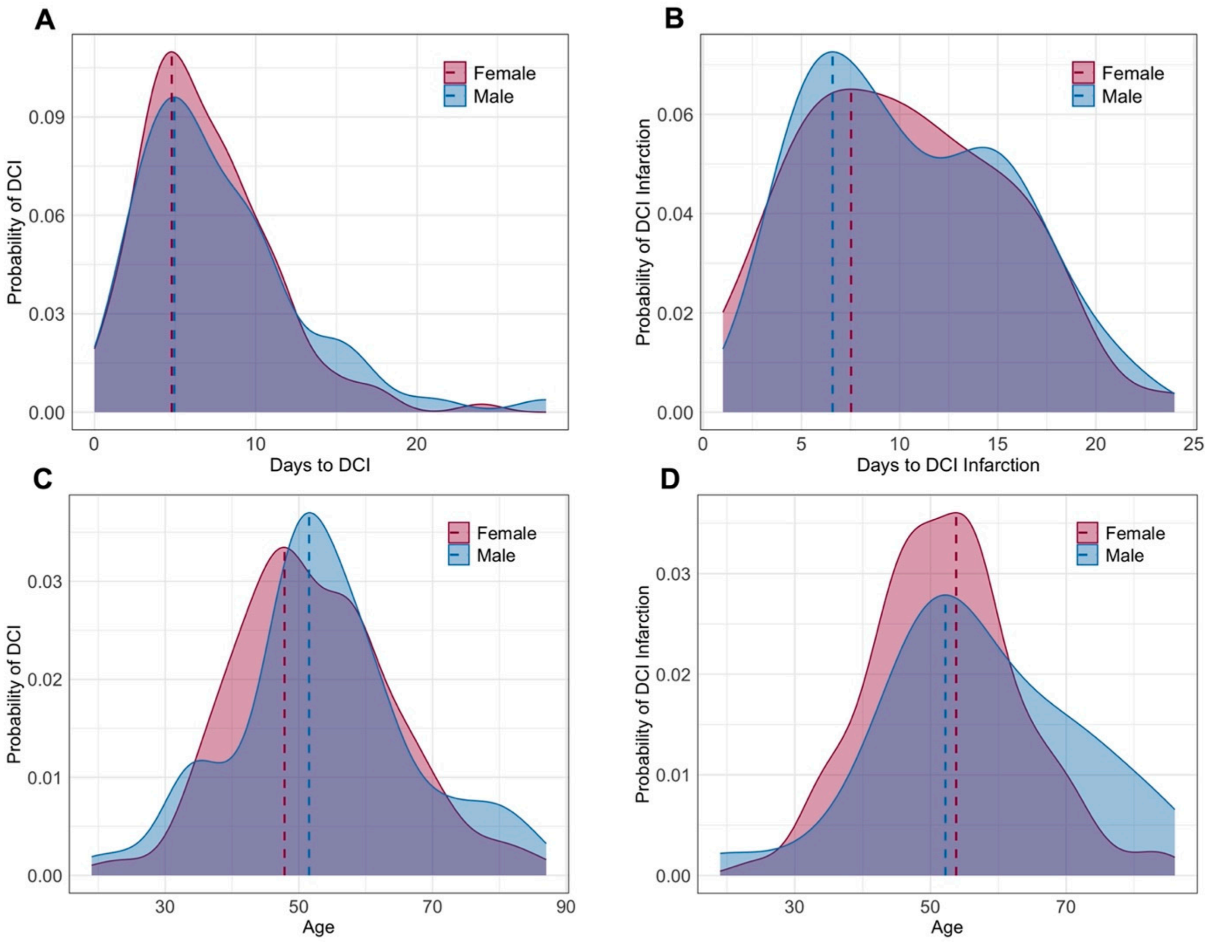

Supplemental Figure S1. Probability density plot of (A) DCI and (B) DCI-related infarction occurring over time (days) after ictus onset (A & B) or in relation to patient age (C & D).

DCI, delayed cerebral ischemia; DCI-infarction, cerebral infarction related to unsuccessful treatment of delayed cerebral ischemia.

Supplemental Tables

Supplemental Table S1

	All<br>n = 650	Female (%)<br>n = 455 (70)	Male (%)<br>n = 195 (30)	univariate p-value
**DCI treatment and outcome - no. (%)**				
ERT available	429 (66.0)	295 (64.8)	134 (68.7)	0.386
DCI occurrence	260 (40.0)	189 (41.5)	71 (36.4)	0.361
DCI lag (days) - median (Q1 to Q2)	6 (4 to 9)	6 (4 to 9)	6 (4 to 10)	0.481
DCI whilst ERT available*	198 (46.2)	142 (48.1)	56 (41.8)	0.264
ERT*	98 (22.8)	66 (22.4)	32 (23.9)	0.825
spasmolysis	86 (13.2)	60 (13.2)	26 (13.3)	0.316
angioplasty	18 (2.8)	10 (2.2)	8 (4.1)	0.346
DCI-related infarction	126 (19.4)	95 (20.9)	31 (15.9)	0.170
DCI-related infarction lag (days)	8 (5 to 10)	8 (5 to 11)	7 (5 to 10)	0.801
infarction volume (ml) - median (Q1 - Q3)	115.0 (36.5 to 279.0)	109.0 (31.6 to 243.0)	154.0 (50.8 to 286.0)	0.670
speech area affected†	33 (26.2)	26 (27.4)	7 (22.6)	0.748
motor area affected	66 (52.3)	50 (52.6)	16 (51.6)	1.0
**Intensive care stay**				
ICU LOS (days) - median (Q1 to Q3)	18 (12 to 31)	17 (13 to 31)	18 (12 to 32)	0.935
hydrocephalus	467 (71.8)	333 (73.2)	134 (68.7)	0.322
pneumonia	232 (35.7)	149 (32.7)	83 (42.6)	0.021
sepsis	94 (14.5)	66 (14.5)	28 (14.4)	1.0
meningitis	72 (11.1)	48 (10.5)	24 (12.3)	0.593
DHC	93 (14.3)	71 (15.6)	22 (11.3)	0.185
hospital LOS (days) - median (Q1 to Q3)	24 (17 to 36)	25 (18 to 36)	23 (16 to 37)	0.393
**Clinical outcome**				
**mRS discharge - no. (%)**				**0.009**
no symptoms	3 (0.5)	2 (0.4)	1 (0.5)	
no significant disability	56 (8.6)	35 (7.7)	21 (10.8)	
slight disability	95 (14.6)	72 (15.8)	23 (11.8)	
moderate disability	96 (14.8)	72 (15.8)	24 (12.3)	
moderate severe disability	124 (19.1)	83 (18.2)	41 (21.0)	
severe disability	139 (21.4)	93 (20.4)	46 (23.6)	
dead / in hospital mortality	137 (21.1)	97 (21.3)	40 (20.5)	
favorable outcome (mRS 0-3)	250 (38.5)	181 (40.0)	69 (35.4)	0.333
**mRS 12 months - no. (%)‡**				0.785
no symptoms	103 (17.9)	71 (15.6)	32 (16.4)	
no significant disability	117 (20.3)	80 (17.6)	37 (19.0)	
slight disability	103 (17.9)	79 (17.4)	24 (12.3)	
moderate disability	37 (6.4)	26 (5.7)	11 (5.6)	
moderate severe disability	47 (8.2)	35 (7.7)	12 (6.2)	
severe disability	27 (4.7)	21 (4.6)	6 (3.1)	
dead	142 (24.7)	98 (21.8)	44 (22.6)	
favorable outcome (mRS 0-3)	360 (62.5)	256 (56.3)	104 (53.3)	0.547
missing	74 (11.3)	45 (9.9)	29 (14.9)	0.090
shunt dependency	138 (21.2)	96 (21.1)	42 (21.5)	0.983

Suppl. Table S1. Specific and non-specific intensive care complications along clinical outcome after 6 and 12 months of follow-up.

DCI, delayed cerebral ischemia; DHC, decompressive hemicraniectomy; ERT, endovascular rescue treatment; ICU, intensive care unit; LOS, length of stay; mRS, modified Rankin scale; Q1, first quartile; Q3, third quartile.

*The proportion of DCI while ERT was available (%) and applied ERT (%) has the total number of patients included when ERT was available in the denominator.

†The proportion of infarct location (%), has the total number of patients with infarction in the denominator.

‡The proportions per outcome category after 12 months (%) has the number of patients with available outcome (n = 576) in the denominator

Supplemental Table S2

**DCI**	**All n = 650**	**DCI n = 260**	**no DCI n = 390**	**univariate p-value**	**OR for DCI**	**95 % CI**	**multivariate p-value**
sex - female / male*	455 (70) / 195 (30)	189 (72.7) / 71 (27.3)	278 (71.3) / 112 (28.7)	0.361	0.774	0.524 to 1.14	0.192
age - yrs. - mean ± SD (range)	55.1 ± 13.2 (19- 90)	52.7 ±12.4 (19 - 87)	56.4 ±13.3	**< 0.001**	0.969	0.955 to .983	**< 0.001**
poor-grade SAH (WFNS 3-5) - no. (%)	269 (41.4)	114 (43.8)	130 (33.3)	0.059	1.55	1.03 to 2.34	**0.038**
mFisher (3-4) - no. (%)	404 (62.2)	190 (73.1)	188 (48.2)	**< 0.001**	2.30	1.51 to 3.52	**< 0.001**
ICH	217 (33.4)	109 (41.9)	99 (25.4)	**< 0.001**	1.27	0.832 to 1.93	0.269
**aneurysm location**				**0.002**			
Acomm	204 (31.4)	100 (38.5)	104 (26.7)	**0.002**	Reference		
ICA	137 (21.1)	60 (23.1)	77 (19.7)	0.356	0.691	0.426 to 1.12	0.133
MCA	170 (26.2)	66 (25.4)	104 (26.7)	0.785	0.502	0.314 to 0.801	**0.004**
BA	49 (7.5)	15 (5.8)	31 (7.9)	0.365	0.547	0.263 to 1.140	0.107
others	90 (13.8)	19 (7.3)	71 (18.2)	**< 0.001**	0.296	0.156 to 0.559	**< 0.001**
max. diameter (mm) - median (Q1 - Q3)	6.0 (4.0 to 8.2)	6 (4 to 8)	6 (4 to 8.8)	0.582			
clipping / endovascular	298 (45.8) / 352 (54.2)	120 (46.2) / 140 (53.8)	178 (45.6) / 212 (54.4)	0.414			
INM available	328 (50.5)	139 (53.5)	189 (48.5)	0.383	0.649	0.396 to 1.06	0.086
ERT available	429 (66.0)	198 (76.2)	231 (59.3)	**< 0.001**	3.42	1.99 to 5.88	**< 0.001**
favorable outcome (mRS 0-3)*	360 (62.5)	125 (21.7)	235 (40.8)	**< 0.001**			
**DCI-related infarction**	**All with DCI n = 260**	**DCI infarction n = 126**	**no DCI infarction n = 134**	**univariate p-value**	**OR for DCI infarction**	**95 % CI**	**multivariate p-value**
sex - female / male*	189 (72.7) / 71 (27.3)	95 (75.4) / 31 (24.6)	94 (70.1) / 40 (29.9)	0.463	0.734	0.394 to 1.37	0.332
age - yrs. - mean ± SD (range)	52.7 ±12.4 (19 - 87)	53.6 ± 12.3 (19 - 86)	51.8 ± 12.8 (19 - 87)	0.258	1.02	0.993 to 1.04	0.167
poor-grade SAH (WFNS 3-5) - no. (%)	114 (43.8)	74 (58.7)	40 (29.9)	**< 0.001**	2.18	1.16 to 4.11	**0.016**
mFisher (3-4) - no. (%)	190 (73.1)	108 (85.7)	82 (61.2)	**< 0.001**	3.25	1.59 to 6.65	**0.001**
ICH	109 (41.9)	57 (45.2)	52 (38.8)	0.303	0.980	0.532 to 1.81	0.948
**aneurysm location**				0.380			
Acomm	100 (38.5)	50 (39.7)	50 (37.3)	0.791	Reference		
ICA	60 (23.1)	26 (20.6)	34 (8.7)	0.448	1.09	0.530 to 2.23	0.818
MCA	66 (25.4)	40 (31.7)	26 (19.4)	**0.032**	0.444	0.217 to 0.911	**0.027**
BA	15 (5.8)	8 (6.3)	7 (5.2)	0.902	0.520	0.217 to .911	0.301
others	19 (7.3)	11 (8.7)	8 (6.0)	0.538	0.661	0.214 to 2.05	0.473
max. diameter (mm) - median (Q1 to Q3)	6 (4.0 to 8.0)	6 (4.0 to 8.0)	6 (4.3 to 9.5)	0.137			
clipping / endovascular	120 (46.2) / 140 (53.8)	50 (39.7) / 76 (60.3)	70 (52.2) / 64 (47.8)	0.094			
INM available	139 (53.5)	54 (42.9)	85 (63.4)	**0.002**	0.306	0.146 to 0.639	**0.002**
ERT available	198 (76.2)	91 (72.2)	107 (79.9)	0.282	1.36	0.600 to 3.09	0.460
favorable outcome (mRS 0-3)*	125 (48.1)	34 (27.0)	91 (67.9)	**< 0.001**			
**one-year outcome**	**All n = 576**	**unfavorable outcome n = 216**	**favorable outcome n = 360**	**univariate p-value**	**OR for unfavorable outcome**	**95 % CI**	**multivariate p-value**

sex - female / male	410 (71.2) / 166 (28.8)	154 (71.3) / 62 (28.7)	256 (71.1) / 104 (28.9)	1.0	0.827	0.528 to 1.30	0.409
age - yrs. - mean ± SD (range)	54.4 ± 12.9 (19- 87)	58.1 ± 13.6 (19 - 87)	52.2 ±12.0 (19 - 83)	**< 0.001**	1.03	1.01 to 1.04	**0.001**
poor-grade SAH (WFNS 3-5) - no. (%)	243 (42.2)	152 (70.4)	90 (25.0)	**< 0.001**	3.35	2.08 to 5.38	**< 0.001**
mFisher (3-4) - no. (%)	354 (61.5)	183 (84.7)	171 (47.5)	**< 0.001**	3.64	2.22 to 5.97	**< 0.001**
ICH	196 (34.0)	101 (46.8)	95 (26.4)	**< 0.001**	1.39	0.871 to .21	0.167
**aneurysm location**				0.250			
Acomm	177 (30.7)	62 (28.7)	115 (31.9)	0.470	Reference		
ICA	127 (22.0)	66 (30.6)	61 (16.9)	**< 0.001**	0.673	0.383 to .18	0.169
MCA	153 (26.6)	41 (19.0)	112 (31.1)	**0.002**	1.08	0.635 to 1.85	0.768
BA	45 (7.8)	15 (6.9)	30 (8.3)	0.659	0.764	0.329 to 1.77	0.532
others	74 (12.8)	32 (14.8)	42 (11.7)	0.335	0.661	0.324 to 1.35	0.255
max. diameter (mm) - median (Q1 to Q3)	6 (4 to 8)	7 (4.7 to 9)	6 (4 to 8)	**0.032**	1.05	0.995 to 1.10	0.080
clipping / endovascular	257 (44.6) / 319 (55.4)	87 (40.3) / 129 (59.7)	170 (47.2) / 190 (52.8)	0.815			
INM available	296 (51.4)	114 (52.8)	182 (50.6)	0.667	0.751	0.422 to 1.34	0.329
ERT available	385 (66.8)	148 (68.5)	237 (65.8)	0.568	1.69	0.911 to 3.12	0.096

Suppl. Table S2. Uni- and multivariate analysis of factors associated with delayed cerebral ischemia (DCI), DCI-related infarction and dichotomized clinical outcome after 12 months. Favorable outcome refers to modified Rankin scale 0-3 and unfavorable outcome to modified Rankin scale 4-6. Multivariate analysis is the result of Generalized Linear Mixed-Effect Modelling applying the availability of INM and ERT as random effects.

Acomm, anterior communication artery; BA, basilar artery; DCI, delayed cerebral ischemia; ERT, endovascular rescue treatment; ICA, internal carotid artery; ICH, intracerebral hemorrhage; INM, invasive neuromonitoring; MCA, middle cerebral artery; mm, millimeter; mFisher, modified Fisher scale; mRS, modified Rankin scale; others, refers to other aneurysm location apart from those mentioned; Q1, first quartile; Q3, third quartile; SAH, aneurysmal subarachnoid hemorrhage; SD, standard deviation; WFNS, World Federation of Neurosurgical Societies aneurysmal subarachnoid hemorrhage grading scale; yrs., years.
